# Atropia *vs.* Morphia

**Published:** 1870-08

**Authors:** Theo. W. Stull

**Affiliations:** Marengo, Ill.


Article VI.—*Atropia* vs. *Morphia*. By Theo. W. Stull, M.D., Marengo, Ill.

As the antidotal powers of Atropia against Opium or any preparations therefrom, is pretty well established, as a fact in therapeutics, I have thought that perhaps the report of an unsuccessful case of my own, might suggest to others, as it has to me, that the failure should more properly be attributed to myself than to a lack of virtue in the medicine.

April 21st, 1870, was called to see Mr. A. V. L., by the druggist, who, a short time before, had sold the man 10 grains of Morphine (Sulphate), and, by the way, not without the requisite amount of caution. I arrived at 4 o'clock, P. M., and found the man lying on the bed, surrounded with friends anxious and importunate that something should be done at once. The patient seemed calm, and complained that we were raising a terrible "rumpus," and did not want to be disturbed. He said he had taken the ten grains at once, somewhere from half an hour to an hour before. He had eaten ravenously several times during the day, and only a short time before taking the dose had eaten an incredibly large dinner at a dining hall. The breath unmistakably showed that he had been drinking freely. I at once gave twenty grains Sulphate of Zinc in one-third glass of water, which he refused to take at first, but made no resistance when it was urged upon him. In ten minutes it was repeated, and twenty grains of Ipecac. added, he then remarking that he was just now getting into a condition to take a nap. Tickling the fauces was of no avail, and a third dose of the Zinc as well—the stupor coming rapidly on, and he was soon entirely insensible. We had no stomach pump, and I now resorted to the Atropia by hypodermic injection. The pupil was contracted to a pin-head in size, respiration seven per minute, pulse 120 and skin blue. Absorption was extremely slow, and it was some little time before the dose of $\frac{1}{48}$ grain Atropia Sulph. was absorbed. It was repeated, until in half an hour one-twelfth grain had been given. The result was that the pupil became dilated to its fullest extent, the respiration rose to twelve per minute, and in this condition he remained till death closed the scene, a few minutes before ten o'clock, in the evening. Dr. Hagar came to my assistance about six o'clock, and we improvised a stomach pump from a large gum elastic catheter and a syringe, but it was of little effect, except that we were able to fill the stomach full of strong hot coffee.

And, now, though the physiological effect of the Atropia upon the pupil was maintained throughout, ought not the dose to have been increased? I think it should, and on a similar occasion would not stop short of three-quarters of a grain of the Atropia, deeming that amount nearer an equivalent to the large amount of Morphine taken in this case. I would not be governed wholly by the physiological effect produced, unless it seemed entirely adequate, soon, but rather by the amount of the opiate taken. There was a marked amelioration of the symptoms upon the pupil becoming dilated.

